# *Notes from the Field*: Investigation of Carbapenemase-Producing Carbapenem-Resistant Enterobacteriaceae Among Patients at a Community Hospital — Kentucky, 2016

**DOI:** 10.15585/mmwr.mm665152a5

**Published:** 2018-01-05

**Authors:** Sae-Rom Chae, Anna Q. Yaffee, Mark K. Weng, D. Cal Ham, Kimberly Daniels, Amanda B. Wilburn, Kimberly A. Porter, Andrea H. Flinchum, Sandra Boyd, Alicia Shams, Maroya S. Walters, Alexander Kallen

**Affiliations:** ^1^Epidemic Intelligence Service, CDC; ^2^Division of Foodborne, Waterborne, and Environmental Diseases, National Center for Emerging and Zoonotic Infectious Diseases, CDC; ^3^Kentucky Department for Public Health; ^4^Division of Healthcare Quality Promotion, National Center for Emerging and Zoonotic Infectious Diseases, CDC; ^5^Division of State and Local Readiness, Office of Public Health Preparedness and Response, CDC.

Carbapenemase-producing carbapenem-resistant Enterobacteriaceae (CP-CRE) express plasmid-encoded carbapenemases, enzymes that inactivate carbapenem antibiotics. They have the potential for epidemic spread through person-to-person transmission and horizontal transfer of resistance mechanisms (*1*,*2*). Typically, CP-CRE are associated with health care exposure. Clinical CRE infections can have mortality rates as high as 50% (*3*); however, the majority of CRE patients are asymptomatic. These asymptomatic colonized patients can serve as a source for transmission to other patients (*4*).

On August 11, 2016, two *Klebsiella pneumoniae* carbapenemase (KPC)–producing CP-CRE isolates from clinical cultures were reported from patients hospitalized at a rural, community hospital in Kentucky; CRE had not been identified previously at this facility. During the next 4 months, an additional 21 CRE isolates were identified from facility patients, resulting in a total of 23 isolates, including 17 *K. pneumoniae,* five *Escherichia coli*, and one *Enterobacter cloacae* isolate. Seventeen (74%) of these isolates were identified through patient screening cultures; the rest were from clinical cultures. Two carbapenemase types were identified through testing of 14 available isolates; 13 produced KPC and one produced New Delhi metallo-β-lactamase. All CP-CRE were *K. pneumoniae* with the exception of two KPC-producing *E. coli.* Pulsed-field gel electrophoresis of these isolates identified three indistinguishable pairs, one of which was the KPC-producing *E. coli* isolates. Medical chart review and patient interviews indicated that the patients from whom each pair had been isolated had exposure to the emergency department or to the same medical-surgical ward, suggesting transmission on these units. Common health care exposures outside the hospital were not identified among the three pairs. Five of 13 interviewed patients reported receipt of health care outside the local area; three might have introduced CP-CRE into the facility, including one patient who was not screened at admission and two who had CRE identified from admission screening. Targeted environmental cultures identified CP-CRE on an emergency department environmental services cart and from the floor sink drain of the involved medical-surgical ward’s environmental services closet.

This investigation suggested CP-CRE in this Kentucky facility was likely attributable to both importation into and transmission within the facility and highlights two points relevant to CP-CRE control. First, demonstration of environmental services cart contamination is notable and suggests a possible role for cleaning equipment in CP-CRE spread. This equipment can move between patient rooms and might not be cleaned regularly. Further investigation is needed to better understand the role of this equipment in transmission of resistant organisms in health care facilities. Second, although CP-CRE has been primarily identified from urban areas, these multidrug-resistant organisms can be introduced into rural areas by patients with exposure to health care in higher CP-CRE–prevalence areas, resulting in local transmission. Facilities in lower CP-CRE–prevalence areas that treat patients who also access care in higher prevalence areas should be aware of this risk. Recommendations to this facility included initiation of CRE surveillance for patients at high risk (e.g., patients with health care exposures during the past year in areas with known higher CP-CRE prevalence); reinforcement of daily and terminal cleaning practices by the environmental services team, including daily cleaning of environmental services carts; and working with facilities in its patient-sharing network to implement a regional CP-CRE prevention strategy (*5*,*6*).
